# Gender differences in patterns of drug use and sexual risky behaviour among crack cocaine users in Central Brazil

**DOI:** 10.1186/s12888-017-1569-7

**Published:** 2017-12-28

**Authors:** Rafael Alves Guimarães, Vivianne de Oliveira Landgraf de Castro, Sandra Maria do Valle Leone de Oliveira, Andréa Cristina Stabile, Ana Rita Coimbra Motta-Castro, Megmar Aparecida dos Santos Carneiro, Lyriane Apolinário Araujo, Karlla Antonieta Amorim Caetano, Marcos André de Matos, Sheila Araujo Teles

**Affiliations:** 1 Institute of Tropical Pathology and Public Health, Federal University of Goiás, Goiânia, Goiás Brazil; 2 Federal University of Mato Grosso do Sul, Campo Grande, Mato Grosso do Sul Brazil; 3 Oswaldo Cruz Foundation, Campo Grande, Mato Grosso do Sul Brazil; 4 Faculty of Nursing, Federal University of Goiás, Goiânia, Goiás Brazil

**Keywords:** Crack cocaine users, Gender, Sexual risky behaviours, Syphilis

## Abstract

**Background:**

The aim of this study was to compare sociodemographic characteristics, patterns of drug use, and risky sexual behaviour among female and male users of crack cocaine.

**Methods:**

Between 2012 and 2013, we conducted a cross-sectional study of 919 crack cocaine users (783 men and 136 women) in Central Brazil using face-to-face interviews. Blood samples were collected to test for syphilis. The Chi-Square Automatic Interaction Detector (CHAID) was used to explore the differences between genders. We implemented two models: the first model included previous incarceration and variables related to patterns of drug use, and the second model included variables related to sexual risky behaviours and syphilis exposure.

**Results:**

Women consumed more crack cocaine than men on a regular basis; however, poly-drug use was more common among men. More women than men reported exchanging sex for money and/or drugs and inconsistent condom use during sexual intercourse; women also reported more sexual partners. In addition, the frequency of sexual violence was higher for women than men. A higher proportion of women than men were positive for syphilis (27.2% vs. 9.2%; *p* < 0.001). The CHAID decision tree analysis identified seven variables that differentiated the genders: previous incarceration, marijuana use, daily crack cocaine consumption, age at first illicit drug use, sexual violence, exchange of sex for money and/or drugs, and syphilis exposure.

**Conclusion:**

Our findings demonstrate a difference in patterns of crack cocaine consumption and sexual risky behaviours between genders, thus indicating a need for gender-specific interventions in this population.

## Background

The consumption of cocaine and crack cocaine are serious public health problems, especially in Latin American countries such as Brazil [1]. These substances have been associated with high morbidity and mortality rates due to violence, chemical dependence, chronic non-communicable diseases, and infectious diseases, especially sexually transmitted infections (STIs) [2, 3]. Between 14 and 21 million people (0.4% of the world’s population) are estimated to use cocaine globally, with the highest prevalence in North and South America [1]. Brazil has one of the highest prevalence rates of cocaine and crack cocaine (“crack”) use; thus, it is a major consumer market for cocaine [4]. A multicentre study conducted in Brazil estimated that 0.81% of the Brazilian population (370,000 individuals) regularly uses crack or similar products (cocaine base paste, *merla*, and oxi). Most crack consumers are young adult men, non-white and with low education. Most are involved in criminal offences [5]. Further, crack consumption has been an important cause of hospitalisation in the context of drug addiction [6].

Some studies have shown gender differences among crack users regarding patterns of drug consumption, sexual risky behaviours, and STIs prevalence [1, 7–11]. Globally in general, men have a higher prevalence of poly-drug use (e.g., marijuana and crack) when compared to women [1]. Women are more vulnerable to heavy consumption, relapse after treatment abandonment, and mental disorder comorbidities [1, 9]. In addition, a multicentre study carried out in the United States of America showed that women engage in more risky behaviours than men, especially exchanges of sex for money and/or drugs, and inconsistent condom use during sexual intercourse [12]. These behaviours increase the STIs prevalence rate (e.g., Human immuno deficiency virus [HIV] and syphilis infections) in the subpopulation of female compared to male crack users [8, 10]. Thus, they should be considered by public health managers when planning strategies for the prevention and control of STIs in this population.

In Brazil, the use of crack is a true epidemic with multiple consequences for healthcare and society, including medical and psychiatric morbidities, premature deaths, and violent behaviours [4, 7]. However, studies of gender differences among crack users in this country are still scarce. To our knowledge, the only investigation carried out in Brazil included few women (*n* = 35), which limited its power to detect gender differences [7]. Thus, our study aimed to compare social and demographic characteristics, patterns of drug use, risky sexual behaviours, and serological status for syphilis between female and male crack users in Central Brazil which is an important corridor for drug trafficking in South America [1]. In addition, we used decision tree analyses to identify patterns of drug use and sexual risks between genders.

## Methods

### Study design, location, and population

Between 2012 and 2013, we conducted a cross-sectional study of crack users who were being treated for detoxification in two large cities of Central Brazil: Goiânia (population of 1302.001 inhabitants) and Campo Grande (population of 786,797 inhabitants). In Goiânia, participants were recruited at a hospital for patients with mental disorders. In Campo Grande, they were recruited from five rehabilitation centres for drug dependence. The eligibility criteria were as follows: age ≥ 18 years, on treatment for detoxification, and self-reported crack use before hospitalisation. Individuals were excluded if they displayed mental confusion and/or aggressive behaviour. Individuals were considered as mentally confused if they were disoriented and were unable to respond to questions clearly. Negative attitudes such as anger manifestation, and verbal or physical threats were considered as aggressive behaviour [13].

### Data collection

The participants were recruited sequentially from a patient list for each health service. Participation was voluntary, and users did not receive any type of compensation. The interviews were conducted face to face in a private setting by members of the research team, who were previously trained to use a standardised instrument about sociodemographic characteristics, crack cocaine and other psychoactive substance use, and sexual risk behaviours. The instrument was based on a questionnaire used in the National Survey on the Profile of Crack Users conducted in Brazil [5], and adapted to use in institutionalized crack users. After the interview, a 10-mL blood sample was collected from each participant for the detection of anti-*Treponema pallidum* antibodies (immunoglobulin M and immunoglobulin G) by enzyme-linked immunosorbent assay (ELISA; Eti-Treponema Plus; DiaSorin S.p.A., UK).

### Statistical analysis

Data were analysed using STATA software, version 14.0. Quantitative variables are presented as median and interquartile range (IQR). Qualitative variables are presented as absolute and relative frequencies. Pearson’s chi-squared test for qualitative variables and Wilcoxon–Mann–Whitney test for quantitative variables were performed to determine the differences between proportions in bivariate analysis. In addition, two different models used the Chi-Square Automatic Interaction Detector (CHAID) [14, 15] to explore differences between genders. The gender (male or female) was considered a variable of outcome. The first model included previous incarceration and variables related to patterns of drug use (Model 1). The second model included variables related to sexual risk behaviours and syphilis exposure (Model 2). The nodes of CHAID were thus defined: (i) root node: node that encompassed the dependent variable gender; (ii) parent’s node: node or algorithm that divides the dependent variable into two or more categories. It corresponds to the category that most influences the dependent variable; (iii) child node: categories found below the parent’s node, and (iv) terminal node: the last category of CHAID. It corresponds to the variable with less importance on the dependent variable [15]. We considered *p* values <0.05 to be statistically significant.

### Ethical considerations

This study was approved by the Human Research Ethics Committee of the Clinical Hospital of the Federal University of Goiás (approval number: 117) and the Ethics Committee of the Federal University of Mato Grosso do Sul (approval number: 438,253). Written consent was obtained from all participants.

## Results

A total of 783 (85.2%) men and 136 (14.8%) women participated in the study. Table 1 presents the characteristics of the study participants and the prevalence of syphilis by gender. There were no significant differences between men and women regarding age, low education, marital status, race/ethnicity, and history of homelessness (*p* > 0.05). The median age of the participants was 30 years (IQR: 12). The majority of participants had low education (62.0%), were single (77.7%), and were non-white (69.3%). In all, 17.5% were homeless in the previous six months.

**Table 1:** Characteristics of crack users in Central Brazil, by gender

Variables	Total	Men	Female	*p* ^*g*^
Sociodemographic characteristics (*n* = 919)				
Age (years), median (IQR)^a^	30.0 (12.0)	31.43 (8.9)	30.60 (8.4)	0.506
Low education^b^ , *n* (%)	570 (62.0)	484 (61.8)	86 (63.2)	0.753
Single marital status, *n* (%)	713 (77.6)	612 (78.3)	101 (74.3)	0.302
Non-white race/ethnicity, *n*(%)	637 (69.3)	541 (69.1)	96 (70.6)	0.727
History of homelessness^c^, *n* (%)	161 (17.5)	133 (17.0)	28 (20.6)	0.308
Previous incarceration^d^ (*n* = 919); *n* (%)	458 (49.8)	415 (53.0)	43 (31.6)	**< 0.001**
Pattern of crack/similar and psychoactive substances use (*n* = 919)				
Age at first illicit drug use, median (IQR)^a^	16 (6)	16 (5)	18 (9)	**0.001**
Duration of crack/similar use (months), median (IQR)^a^	24.0 (52.0)	24.0 (52.0)	36.0 (50.7)	0.589
Daily crack consumption, *n* (%)	551 (60.0)	455 (58.1)	96 (70.6)	**0.006**
Sharing of pipe^c^, *n* (%)	645 (70.2)	547 (70.0)	98 (72.0)	0.612
Smoking mixed crack and tobacco^c^, *n* (%)	377 (41.1)	334 (42.7)	43 (31.9)	**0.018**
Smoking mixed crack and marijuana^c^, *n* (%)	397 (43.2)	350 (44.7)	47 (34.8)	**0.032**
Alcohol^c^ , *n* (%)	676 (73.6)	581 (74.2)	95 (69.9)	0.288
Marijuana^c^, *n* (%)	586 (63.8)	516 (65.9)	70 (51.5)	**0.001**
Intranasal cocaine^c^, *n* (%)	503 (54.7)	442 (56.4)	61 (44.9)	**0.012**
Injecting drugs^d^, *n* (%)	93 (10.1)	77 (9.8)	16 (11.9)	0.473
Sexual behaviours				
Homosexual orientation (*n* = 814), *n* (%)	98 (12.0)	82 (11.9)	16 (12.6)	0.833
Sexual violence^d^ (*n* = 914), *n* (%)	115 (12.6)	64 (8.2)	51 (38.1)	**< 0.001**
Exchanging sex for money and/or drugs^c^ (*n* = 859), *n* (%)	177 (20.6)	122 (16.8)	55 (42.0)	**< 0.001**
Number of sexual partners (*n* = 500)^c^, median (IQR)^a^	3.0 (4.0)	3.0 (2.0)	4.0 (9.0)	**0.011**
Any unprotected sexual intercourse with steady partner^c^ (*n* = 516), *n* (%)	431 (83.5)	346 (83.0)	85 (85.9)	0.487
Any unprotected sexual intercourse with casual partner^c^ (*n* = 506), *n* (%)	301 (59.1)	259 (56.5)	42 (59.2)	0.951
Any unprotected anal intercourse^c^ (*n* = 471), *n* (%)	311 (66.0)	272 (64.9)	39 (75.0)	0.148
STI^e^ history^c^ (*n* = 903), *n* (%)	258 (28.6)	224 (29.1)	34 (25.6)	0.406
Syphilis prevalence (+)^f^ (*n* = 919), *n* (%)	109 (11.9)	72 (9.2)	37 (27.2)	**< 0.001**

^a^Interquartile range

^b^Defined as elementary and middle schools (completed or not)

^c^Previous six months

^d^Lifetime

^e^Sexually transmitted infections

^f^ELISA positivity

^g^Pearson’s chi-squared (qualitative variables) or Wilcoxon–Mann–Whitney (quantitative variables)

Half of the participants reported previous incarceration, with more men having this experience than women (53.0% vs. 31.6%; *p* < 0.001). Men began their illicit drug use earlier in life than women (median age: 16 years vs. 18 years, respectively; *p* = 0.001); however, they reported less compulsive use than women (58.1% vs. 70.6%; *p* = 0.006). Most participants reported the use of multiple substances in the previous six months, such as alcohol (73.6%), marijuana (63.8%), and intranasal cocaine (54.7%); only 10.1% reported previous injection drug use. Smoking mixtures of crack and tobacco or crack and marijuana was reported by 41.1% and 43.3% of participants, respectively.

We found gender differences in patterns of use of these substances. Male crack users were more likely than females to report the intranasal use of cocaine (56.4% vs. 44.9%, respectively; *p* = 0.001) and marijuana (65.9% vs. 51.5%, respectively; *p* = 0.012). Furthermore, men were more likely than women to report smoking a mixture of crack and tobacco (42.7% vs. 31.9%, respectively; *p* = 0.018) or crack and marijuana (44.7% vs. 34.8%, respectively; *p* = 0.032; Table 1).

Female crack users were more likely than males to exchange sex for money and/or drugs (42.0% vs. 16.8%, respectively; *p* < 0.001) and experience sexual violence (38.1% vs. 8.2%, respectively; *p* < 0.001). Furthermore, the median number of sexual partners in the previous six months was greater for women than men (4.0 vs. 3.0; *p* = 0.001). Unprotected sexual intercourse occurred frequently in both genders. However, this behaviour was more common with a regular sexual partner and for anal sex (Table 1).

Approximately one-third of participants (28.6%) reported at least one STI episode in their lifetime. Furthermore, 11.9% (95.0% confidence interval [CI]: 9.9–14.1%) were found positive for anti-*Treponema pallidum* by ELISA. The proportion of women who were positive was three times higher than of men who were positive: 27.2% (95.0% CI: 20.4–35.2%) vs. 9.2% (95.0% CI: 7.4–11.4; *p* < 0.001; Table 1).

**Fig. 1:**
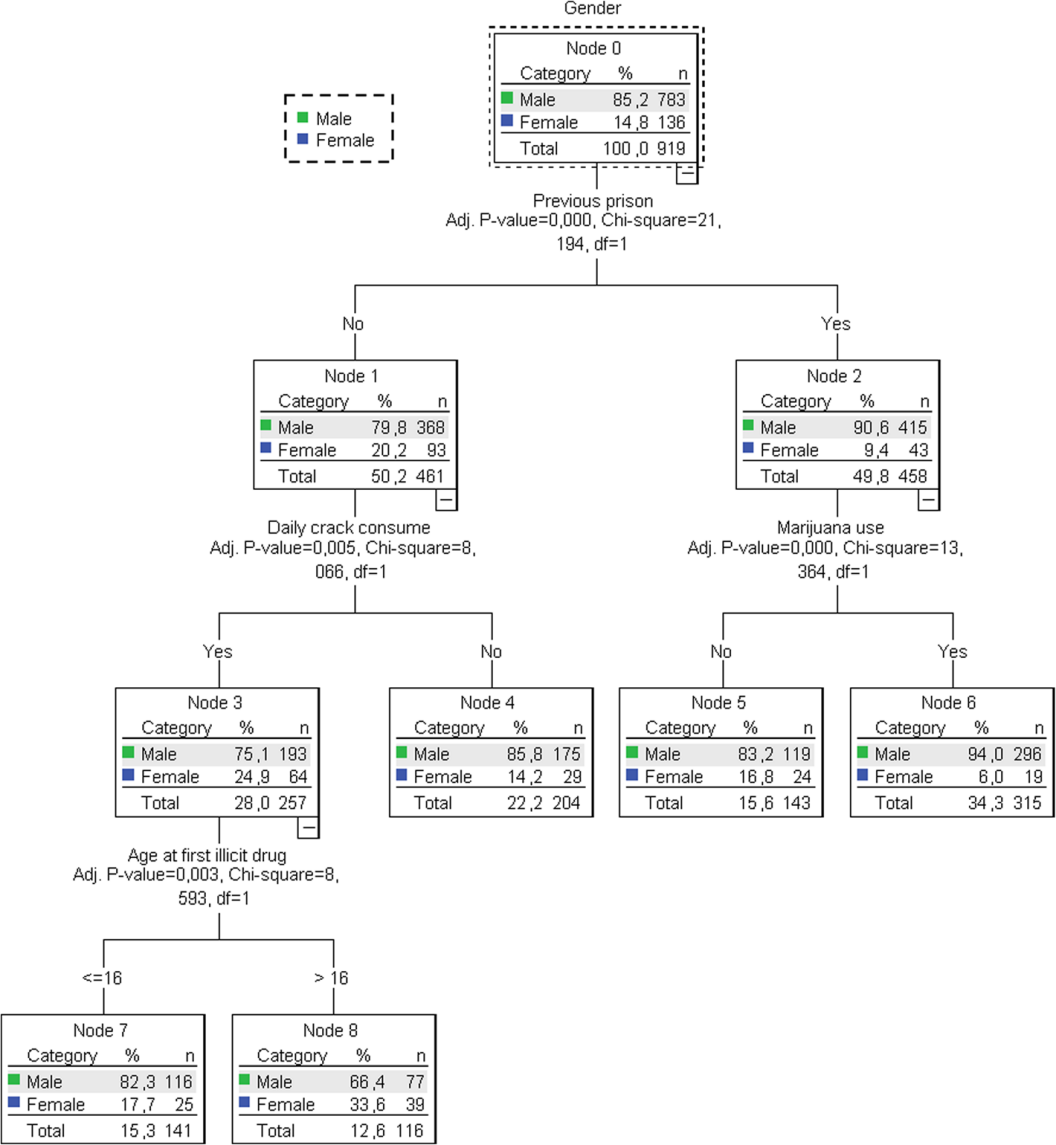
Results of chi-squared automatic interaction detector (CHAID) model for previous prison and pattern of drug use in crack users in Central Brazil

The results of the CHAID analysis for previous incarceration and patterns of drug use (Model 1) are shown in Fig. 1. The dependent variable included the gender (root node). Model 1 included six significant variables on bivariate analysis: previous incarceration, age at first illicit drug use, daily crack consumption, smoking a mixture of crack and tobacco, smoking a mixture of crack and marijuana, marijuana use, and intranasal use of cocaine. This model consisted of four levels and eight nodes. The most important variable differentiating men and women was previous incarceration (*p* < 0.001), which represents the parent’s node. Among individuals with this characteristic, marijuana use (*p* < 0.001) and daily crack consumption (*p* < 0.001) were the discriminating factors between genders (*p* < 0.001). These variables constituted the child nodes of model 1. Furthermore, age at first illicit drug use was the variable that discriminated the genders (*p* = 0.003), constituting the terminal node of CHAID. The estimated error of risk in Model 1 was 0.148 and the standard error was 0.012.

**Fig. 2:**
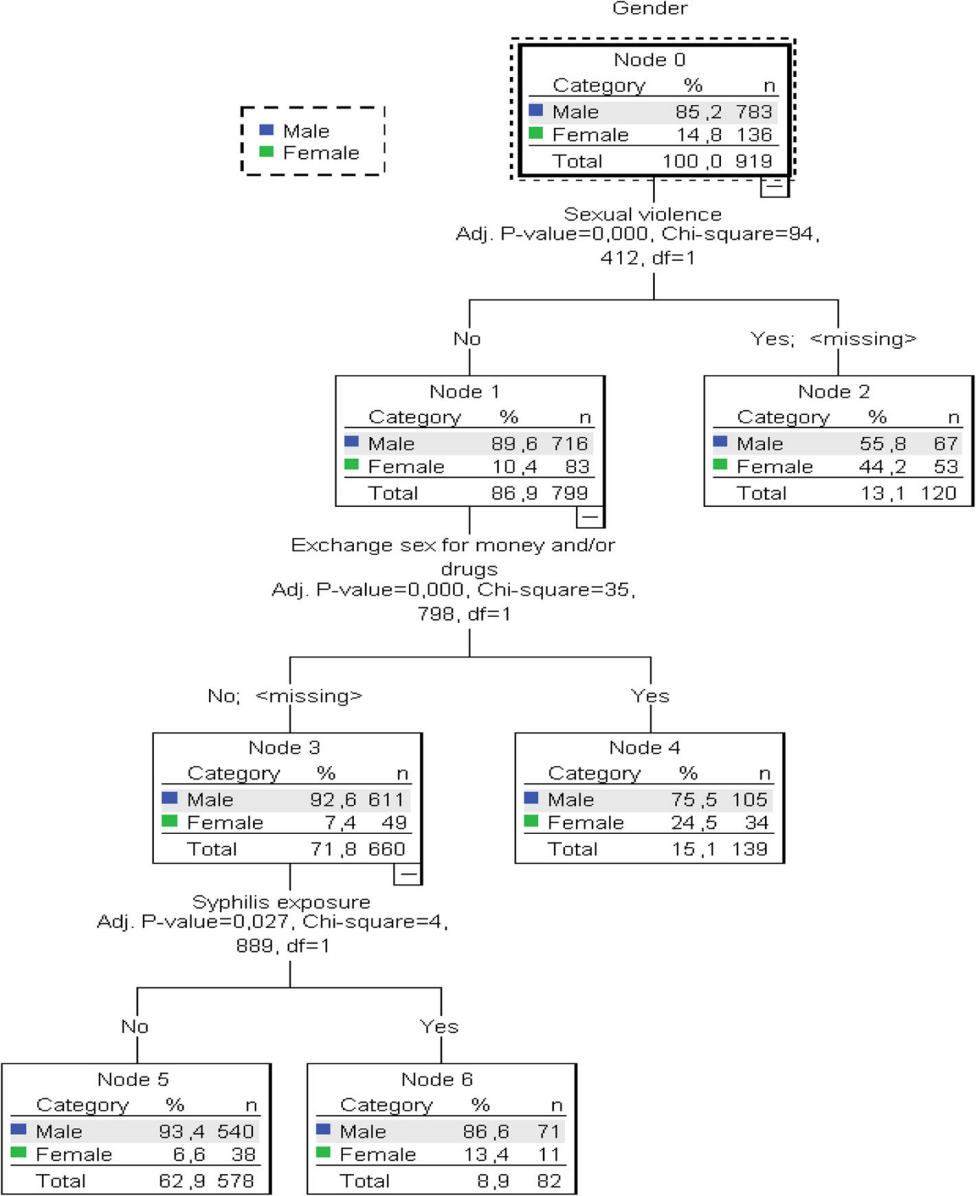
Results of chi-squared automatic interaction detector (CHAID) model sexual risk and syphilis prevalence in crack users in Central Brazil

The results of the CHAID analysis for model 2 are shown in Fig. 2. The dependent variable included the gender (root node). Three variables were included in the analysis: sexual violence, exchange of sex for money and/or drugs, and syphilis exposure. The number of sexual partners was excluded from the CHAID analysis because many of these values were missing. Model 2 consisted of four levels and six nodes. Three variables differentiated the genders after adjustment: sexual violence (parent’s node), exchange of sex for money and/or drugs (child node), and syphilis exposure (terminal node), all of which were found in higher proportions in women than men. The estimated error of risk in Model 2 was 0.148 and the standard error was 0.012.

## Discussion

Our study presents the first data on differences in the patterns of consumption of illicit drugs, risky sexual behaviours, and syphilis exposure in crack users in Central Brazil. We found no differences in sociodemographic characteristics between genders for crack cocaine users in Central Brazil. However, as also observed by Bertoni et al., [7], a large difference was found in the pattern of drug use and sexual risk behaviours.

The median age at first illicit drug use by our participants was 16 years (IQR: 6 years). Furthermore, men first used illicit drugs at an earlier age than women (16 years vs. 18 years, respectively; *p* < 0.001). Other studies have also shown that women start using drugs at a later age than men [16–19]. Gender-related differences in age at onset of drug use are possibly due to men having increased access to illicit substances and a lesser perception of risks related to substance use compared with women [16, 20].

Our findings have some implications for public health. First, the initiation of drug use during early adolescence has been associated with increased dependence and drug abuse [21, 22] and an increased risk of STI acquisition, including HIV [23]. Thus, drug use at an early age can contribute to the overall burden of substance-related illnesses. Furthermore, the results of this study suggest that men have opportunity to access illicit drugs at an earlier age than women. Therefore, interventions and actions to prevent drug use should be intensified for adolescent males.

In the context of crack addiction, poly-drug consumption seems to be a common behaviour [7, 11, 24, 25]. In fact, the majority of our participants reported this behaviour, although it seems to be more frequent among males. A previous investigation of 160 crack users from two large cities in Southern and North-eastern Brazil showed a higher proportion of alcohol (55.7% vs. 25.7%), marijuana (71.5% vs. 51.4%), and intranasal cocaine (45.8% vs. 11.4%) use among men compared with women [7]. These behaviours (except for alcohol use) were also found in our participants from Central Brazil. In addition, the CHAID decision tree model has also demonstrated marijuana consumption as a variable that differentiates the genders. The simultaneous consumption of substances is a risk factor for chemical dependence, the development of psychological disorders (e.g., depression and anxiety), and physical illnesses [26, 27]. Thus, our findings suggest that these related problems of substance use are more likely to occur in men than women.

Studies in humans and animal models have shown that females are more vulnerable to heavy drug use and recidivism compared with males [20, 28]. The oestrogen hormone is believed to play a role in the abuse and heavy consumption of illicit drugs by women [29]. Our findings contribute to this assumption. Compulsive drug consumption was greater among women, with 70.6% of women and 52.0% of men reporting daily crack consumption, results that were supported by the CHAID analysis.

Smoking crack mixed with tobacco and/or marijuana in the form of a cigarette was frequently reported by our participants (~40%); however, this behaviour was more frequent among men in the bivariate analysis (*p* < 0.05). In qualitative studies using in-depth interviews with crack users, the concurrent use of crack and marijuana seems to diminish the undesirable effects of crack (e.g., cravings), and consequently the risk behaviours and violence related to its consumption. Nevertheless, clinical investigations are necessary to elucidate the real role of this strategy in reducing the harm associated with crack use [30, 31].

Unprotected sexual intercourse was the most frequently reported risky sexual behaviour among our participants of either gender. Unprotected sexual intercourse with a steady partner, unprotected anal intercourse, and unprotected sexual intercourse with casual partners were also reported. These behaviours were also found in a previous study conducted among drug users in the United States of America, although these authors found a higher proportion of unprotected sexual intercourse with steady partners occurring among female drug users compared with males (82.4% vs 75.4%; *p* = 0.016) [12]. In general, long-term relationships create a sense of security and trust that precludes and/or reduces condom use. A previous study investigated intimacy and its role in relationship management among 73 HIV drug users and their partners. According to this investigation, in the context of HIV, unprotected sex between steady partners strengthens the relationship [32].

The exchange of sex for money and/or drugs and sexual violence were more frequently reported among females than males. In addition, the CHAID model showed that these key sexual risk variables differentiated the genders, ratifying the greater social vulnerability of female crack users compared with males [25, 33–35]. Thus, in addition to intrinsic biological characteristics, these factors may increase the susceptibility of women to STIs, which may contribute to the large disparity of syphilis prevalence between genders found in our investigation. In fact, syphilis prevalence was three times greater in women compared to men in our study.

This study has some limitations. Firstly, all information regarding patterns of illicit drug use and risk behaviours was based on self-reports, which may underestimate the prevalence of certain risk behaviours (e.g., exchanging sex for money and/or drugs). Thus, the data may be subject to memory bias and reported according to social desirability. Secondly, the non-probabilistic sample and the inclusion of subjects from only institutions for the treatment of chemical dependence may not allow the results to be generalised to crack users from other geographical locations. However, the sociodemographic characteristics of our participants were similar to those of other crack users in Brazil, suggesting external validity [5]. Thirdly, the sample of women was relatively small, which may have reduced the analytical power of the study.

## Conclusion

The present investigation included a large sample of crack users from a region of the world that is the major corridor for illicit drug trafficking, and shows important differences between males and females in the context of crack consumption. According our findings, the pattern of illicit drug consumption varies between genders, and this should be considered in treatment programmes for dependence. Further, this difference also should be considered when health managers plan STI prevention programmes and harm reduction such that the interventions may be more effective. Finally, our investigation represents the starting point for future research on the gender differences among crack users which can elucidate the role of gender concerning heavy crack use, poly-drug use, and sexual risk behaviours.

## Abbreviations

CHAID: The Chi-Square Automatic Interaction Detector
ELISA: Enzyme-linked immunosorbent assay
HIV: Human immunodeficiency virus
IQR: Interquartile range
STIs: Sexually transmitted infections
